# Supplementary material for: India may need an additional metric to assess the endemicity of malaria in low surveillance districts
Source: PLOS Glob Public Health. 2022 Nov 10;2(11):e0000326. doi: 10.1371/journal.pgph.0000326 (PMC10021988; doi:10.1371/journal.pgph.0000326)
Supplement: S1 Text — (DOCX) [file pgph.0000326.s001.docx]

**S1 Text**

**Table A: Top twenty districts from low surveillance (ABER<5) states of India where the difference between API and SPR is considerable**

| S.No. | State | District | Med. ABER  (2017-19) | Med. API  (2017-19) | Med. SPR  (2017-19) |
| --- | --- | --- | --- | --- | --- |
| 1 | Bihar | Muzaffarpur | 0.01 | 0 | 7.67 |
| 2 | Bihar | Begusarai | 0.02 | 0 | 4.68 |
| 3 | Bihar | Jamuie | 0.5 | 0.17 | 3.78 |
| 4 | Bihar | East Champaran | 0 | 0 | 3.25 |
| 5 | Bihar | Samastipur | 0.01 | 0 | 3.12 |
| 6 | Bihar | Kaimur | 0.37 | 0.09 | 2.71 |
| 7 | Bihar | Gaya | 0.42 | 0.15 | 2.55 |
| 8 | Bihar | Sheohar | 0.01 | 0 | 2.38 |
| 9 | Bihar | Buxar | 0.1 | 0.02 | 2.32 |
| 10 | Bihar | Munger | 0.59 | 0.09 | 2.33 |
| 11 | Bihar | Madhubani | 0.11 | 0.01 | 1.67 |
| 12 | Bihar | Araria | 0.05 | 0 | 1.14 |
| 13 | Bihar | Lakhisarai | 1.16 | 0.06 | 1.15 |
| 14 | Jammu & Kashmir | Jammu urban | 0.28 | 0.03 | 1.23 |
| 15 | Lakshadweep | Lakshadweep | 0.7 | 0.09 | 1.18 |
| 16 | Uttar Pradesh | Hardoi | 1.64 | 0.48 | 2.65 |
| 17 | Uttar Pradesh | Farrukhabad | 2.1 | 0.5 | 2.38 |
| 18 | Uttar Pradesh | Allahabad | 1.97 | 0.35 | 1.82 |
| 19 | Uttar Pradesh | Bhadohi | 2.3 | 0.37 | 1.61 |
| 20 | Uttar Pradesh | Sitapur | 0.92 | 0.13 | 1.33 |

**Table B: Top twenty districts from moderate surveillance (5≤ABER≤15) states of India where the difference between API and SPR is considerable**

| Sno | State | District | Med_ABER  (2017-19) | Med_API  (2017-19) | Med_SPR  (2017-19) |
| --- | --- | --- | --- | --- | --- |
| 1 | WEST BENGAL | KOLKATA(KMC) | 7.67 | 3.72 | 4.24 |
| 2 | JHARKHAND | GARHWA | 11.78 | 3.87 | 3.47 |
| 3 | TRIPURA | SOUTH TRIPURA | 10.51 | 2.28 | 2.17 |
| 4 | TRIPURA | GOMATI | 7.76 | 2.02 | 2.6 |
| 5 | JHARKHAND | PALAMAU | 8.37 | 2.75 | 3.35 |
| 6 | ODISHA | SUNDERGARH | 12.64 | 1.87 | 1.54 |
| 7 | JHARKHAND | SARAIKELA | 10.05 | 3.10 | 3.09 |
| 8 | MIZORAM | SAIHA | 11.63 | 3.10 | 2.89 |
| 9 | JHARKHAND | KHUNTI | 13.72 | 2.77 | 2.02 |
| 10 | TRIPURA | NORTH TRIPURA | 8.28 | 2.50 | 2.15 |
| 11 | JHARKHAND | CHATRA | 8.87 | 2.48 | 2.78 |
| 12 | ODISHA | MAYURBHANJ | 14.97 | 0.81 | 0.56 |
| 13 | TRIPURA | KHOWAI | 7.26 | 1.70 | 1.93 |
| 14 | MEGHALAYA | SOUTH WEST KHASI HILLS | 5.9 | 0.62 | 1.42 |
| 15 | MEGHALAYA | WEST KHASI HILLS | 5.9 | 0.62 | 1.42 |
| 16 | ODISHA | GANJAM | 10.98 | 2.43 | 2.2 |
| 17 | ASSAM | UDALGURI | 9.37 | 1.24 | 1.33 |
| 18 | JHARKHAND | KODERMA | 6.05 | 1.17 | 2.09 |
| 19 | MADHYA PRADESH | ANUPPUR NEW | 11.9 | 0.76 | 0.7 |
| 20 | JHARKHAND | GUMLA | 8.98 | 1.16 | 1.29 |

**Table C: Top twenty districts from high surveillance (ABER>15) states of India where the difference between API and SPR is considerable**

| Sno | State | District | Med_ABER  (2017-19) | Med_API  (2017-19) | Med_SPR  (2017-19) |
| --- | --- | --- | --- | --- | --- |
| 1 | CHHATTISGARH | Bijapur | 50.3 | 54.21 | 11.18 |
| 2 | CHHATTISGARH | Dantewada (sukma, narayanpur) | 43.08 | 41.74 | 9.51 |
| 3 | CHHATTISGARH | Sukma | 31.33 | 42.9 | 13.86 |
| 4 | CHHATTISGARH | Narayanpur | 32.63 | 31.68 | 10.16 |
| 5 | MEGHALAYA | South garo hills | 25.1 | 32.76 | 12.86 |
| 6 | ODISHA | Malkangiri | 29.32 | 21.34 | 7.21 |
| 7 | ODISHA | Rayagada | 27.19 | 19.62 | 6.72 |
| 8 | ODISHA | phulbani(kandhamal) | 32.2 | 17.38 | 4.96 |
| 9 | MIZORAM | Lawngtlai | 21.76 | 20.83 | 9.61 |
| 10 | TRIPURA | dhalai Tripura | 33.62 | 12.38 | 3.68 |
| 11 | MIZORAM | Mamit | 21.67 | 13.02 | 5.96 |
| 12 | ODISHA | Koraput | 26.36 | 11.36 | 4.3 |
| 13 | ODISHA | Gajapati | 21.87 | 11.01 | 5.04 |
| 14 | CHHATTISGARH | Bastar | 23.56 | 9.89 | 4.19 |
| 15 | A & N ISLANDS | Nicobar | 28.39 | 7.26 | 2.54 |
| 16 | CHHATTISGARH | Kondagaon | 26.06 | 7.27 | 2.63 |
| 17 | ODISHA | Kalahandi | 19.58 | 9.16 | 4.82 |
| 18 | MIZORAM | Lunglei | 16.83 | 10.34 | 6.16 |
| 19 | JHARKHAND | Latehar | 17.55 | 9.78 | 5.65 |
| 20 | CHHATTISGARH | Kanker | 38.7 | 5.1 | 1.28 |


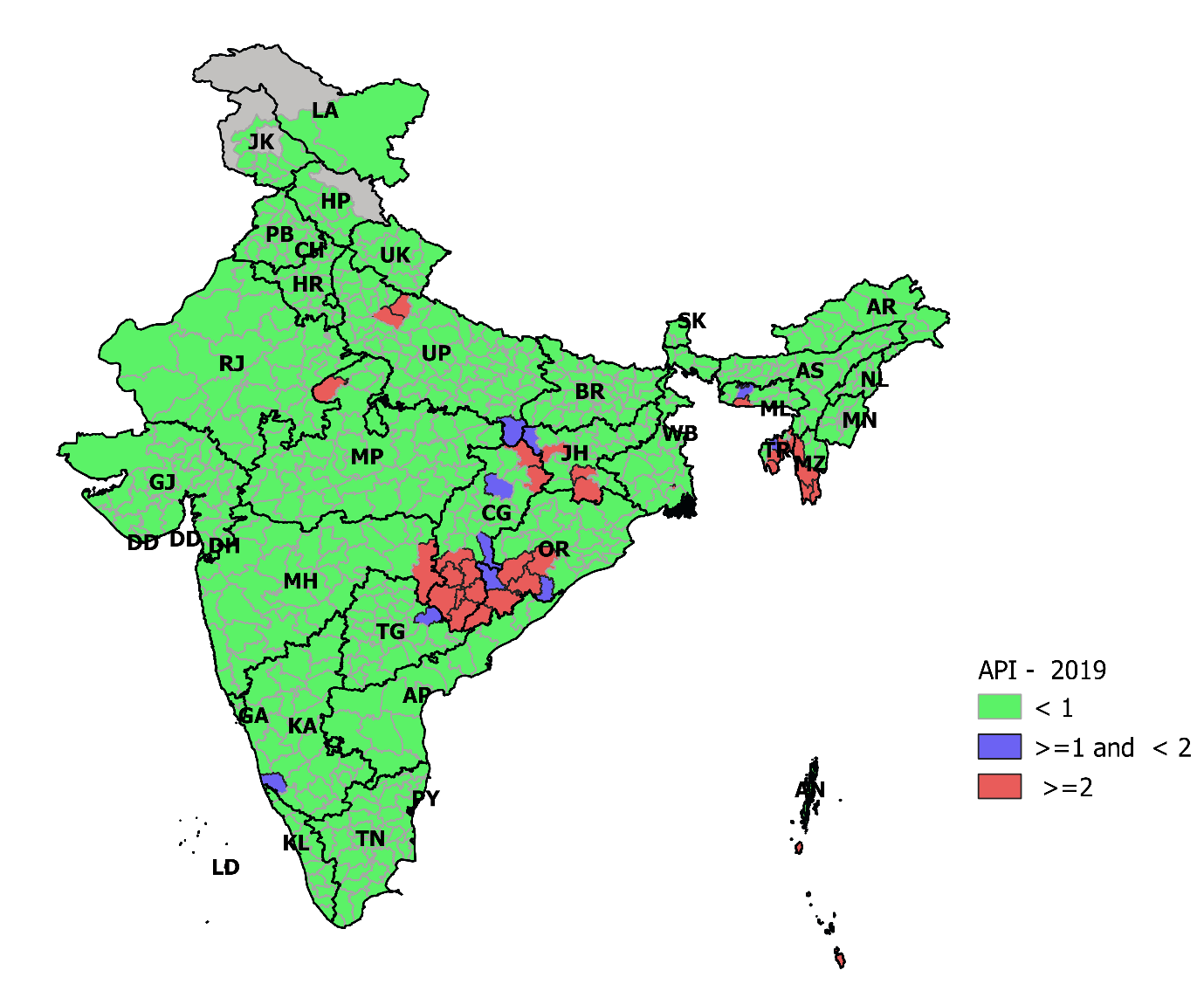


**Fig A: Distribution of Annual Parasite Incidence (API) as per National Strategic Plan-2016-22 (district as a unit) in India**


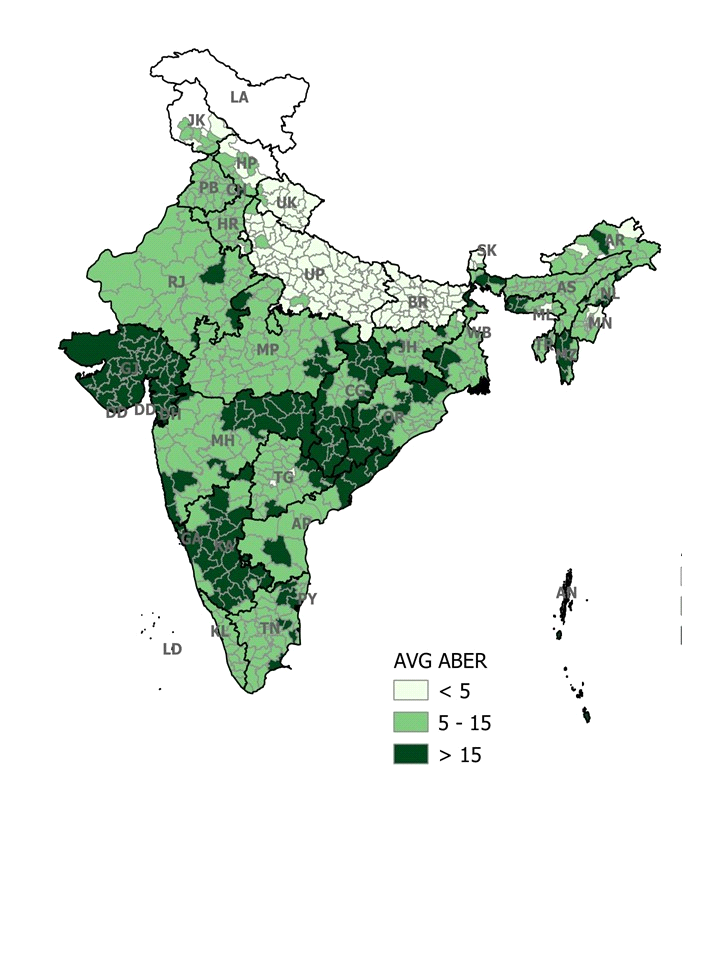


**Fig B: Distribution of Annual Blood Examination Rate (ABER) (district as a unit) in India**

AVG ABER: Average ABER (2017-2019)
