# Supplementary material for: India may need an additional metric to assess the endemicity of malaria in low surveillance districts
Source: PLOS Glob Public Health. 2022 Nov 10;2(11):e0000326. doi: 10.1371/journal.pgph.0000326 (PMC10021988; doi:10.1371/journal.pgph.0000326)
Supplement: Attachment — Submitted filename: Reviewer reply round 4.docx [file pgph.0000326.s002.docx]

Dear Editor team

We thank you for your comments and inputs on our manuscript entitled, "India may need an additional metric to assess the endemicity of Malaria and plan its elimination” **(Ref no PGPH-D-22-00177)***.* We have now addressed each of the comments fully. The comments (in black) and our responses (in blue) are below.

1. Please note that PLOS ONE is unable to publish previously copyrighted maps or satellite images, or images created using proprietary data. For these reasons, we cannot publish images generated by software which copyrights their output (such as Google Maps, Street View, and Earth). In order to use these images in your submission, we require explicit permission from the copyright owner to publish the figures under the CC BY 4.0 license.
   At this time, please kindly address the following regarding Figure 2 and Figure 4:
   We require specific consent from the copyright holder to publish these images in PLOS ONE, under the CC BY 4.0 license.
   To seek permission from the copyright owner to publish your map figures under the specific Creative Commons Attribution License (CCAL), CC BY 4.0, please contact them with the following text and PLOS ONE Request for Permission form (https://journals.plos.org/globalpublichealth/s/file?id=c4ff/Content%20Copyright%20Permission%20Form.pdf):
   “I request permission for the open-access journal PLOS ONE to publish XXX under the Creative Commons Attribution License (CCAL) CC BY 4.0 (http://creativecommons.org/licenses/by/4.0/). Please be aware that this license allows unrestricted use and distribution, even commercially, by third parties. Please reply and provide explicit written permission to publish XXX under a CC BY license.”
   Please upload the granted permission to the manuscript as a Supporting Information file. In the figure caption of the copyrighted figure, please include the following text: “Republished from [ref] under a CC BY license, with permission from [name of publisher], original copyright [original copyright year].”
   Please note that RightsLink permission forms often impose use restrictions that are incompatible with our CC BY 4.0 license, and we are therefore unable to accept these permissions. For this reason, we strongly recommend contacting copyright holders with the PLOS ONE Request for Permission form.
   If you are unable to obtain permission from the copyright holder, please either A) remove the figure or B) supply a replacement figure that complies with the CC BY 4.0 license.


   Please check copyright information on all replacement figures and update the figure caption with source information.
   The following resources for replacing copyrighted map figures may be helpful:
   USGS National Map Viewer (http://viewer.nationalmap.gov/viewer/)
   USGS Earth Resources Observatory and Science (EROS) Center (http://eros.usgs.gov/#)
   The Gateway to Astronaut Photography of Earth (https://eol.jsc.nasa.gov/)
   Maps at the CIA (https://www.cia.gov/library/publications/the-world-factbook/docs/refmaps.html)
   NASA Earth Observatory (http://earthobservatory.nasa.gov/)
   Landsat ( http://landsat.visibleearth.nasa.gov/)
   Natural Earth ( http://www.naturalearthdata.com/)

**Response:** Here, we would like to bring for your kind consideration that the official shape-files (**base layer**) for India were procured/purchased by the Institute from the **Survey of India, Ministry of Science & Technology**, **Government of India**, under the collaboration of NIMR, India and Oxford University, UK. The shapefiles were procured for both state boundaries and district boundaries. **The receipt for the purchase has been submitted to the journal as supplementary material.** The API, ABER, and SPR data were superimposed on the base shape files to create figures 2 to 4 using Arc GIS software.

Regarding the copyright ownership for publishing the figures under the CC BY 4.0 license, we would like to bring to your notice that **Survey of India does not grant individual permissions**. The Survey of India, Ministry of Science and Technology, Government of India, has issued a notification with guidelines (available on [https://onlinemaps.surveyofindia.gov.in/ GeospatialGuidelines.aspx](https://onlinemaps.surveyofindia.gov.in/%20GeospatialGuidelines.aspx)), that **clearly states that there shall be no requirement for prior approval, permission or license to publish these shape-files once purchased**. Only self-certification may be used for adherence to the guidelines.

**The same was also intimated to the editor, and we are very hopeful you will consider our manuscript this time**

**Regards and Thanks**
