# Supplementary material for: India may need an additional metric to assess the endemicity of malaria in low surveillance districts
Source: PLOS Glob Public Health. 2022 Nov 10;2(11):e0000326. doi: 10.1371/journal.pgph.0000326 (PMC10021988; doi:10.1371/journal.pgph.0000326)
Supplement: Attachment — Submitted filename: Reviewer reply minor comments.docx [file pgph.0000326.s003.docx]

Dear Editor

We thank you and the reviewers for their constructive comments and inputs on our manuscript entitled as, "India may need an additional metric to assess the endemicity of Malaria and plan its elimination” **(Ref no PGPH-D-22-00177R1)***.* We have now addressed each of the comments fully. The comments (in black) and our responses (in blue) are below.

Thank you very much indeed for your consideration of this manuscript, and we will be happy to address any other issues with the paper.

Looking forward to your positive response.

Regards,

Amit Sharma

PGPH-D-22-00177R1
India may need an additional metric to assess the endemicity of malaria in low surveillance districts
PLOS Global Public Health

Dear Dr. Sharma,

Thank you for submitting your manuscript to PLOS Global Public Health. After careful consideration, we feel that it has merit but does not fully meet PLOS Global Public Health’s publication criteria as it currently stands. Therefore, we invite you to submit a revised version of the manuscript that addresses the points raised during the review process.

Please submit your revised manuscript by Nov 16 2022 11:59PM. If you will need more time than this to complete your revisions, please reply to this message or contact the journal office at globalpubhealth@plos.org. When you're ready to submit your revision, log on to https://www.editorialmanager.com/pgph/ and select the 'Submissions Needing Revision' folder to locate your manuscript file.

Please include the following items when submitting your revised manuscript:

- A rebuttal letter that responds to each point raised by the editor and reviewer(s). You should upload this letter as a separate file labeled 'Response to Reviewers'.
- A marked-up copy of your manuscript that highlights changes made to the original version. You should upload this as a separate file labeled 'Revised Manuscript with Track Changes'.
- An unmarked version of your revised paper without tracked changes. You should upload this as a separate file labeled 'Manuscript'.

Guidelines for resubmitting your figure files are available below the reviewer comments at the end of this letter.

We look forward to receiving your revised manuscript.

Kind regards,

Louisa Alexandra Messenger, MSc, PhD
Academic Editor
PLOS Global Public Health

Journal Requirements:

Please review your reference list to ensure that it is complete and correct. If you have cited papers that have been retracted, please include the rationale for doing so in the manuscript text, or remove these references and replace them with relevant current references. Any changes to the reference list should be mentioned in the rebuttal letter that accompanies your revised manuscript. If you need to cite a retracted article, indicate the article’s retracted status in the References list and also include a citation and full reference for the retraction notice.

Additional Editor Comments (if provided):


[Note: HTML markup is below. Please do not edit.]

Reviewers' comments:

Reviewer's Responses to Questions

**Comments to the Author**

1. If the authors have adequately addressed your comments raised in a previous round of review and you feel that this manuscript is now acceptable for publication, you may indicate that here to bypass the “Comments to the Author” section, enter your conflict of interest statement in the “Confidential to Editor” section, and submit your "Accept" recommendation.

Reviewer #2: (No Response)

2. Does this manuscript meet PLOS Global Public Health’s [publication criteria](https://journals.plos.org/globalpublichealth/s/criteria-for-publication)? Is the manuscript technically sound, and do the data support the conclusions? The manuscript must describe methodologically and ethically rigorous research with conclusions that are appropriately drawn based on the data presented.

Reviewer #2: Yes

3. Has the statistical analysis been performed appropriately and rigorously?

Reviewer #2: (No Response)

4. Have the authors made all data underlying the findings in their manuscript fully available (please refer to the Data Availability Statement at the start of the manuscript PDF file)?

The [PLOS Data policy](https://journals.plos.org/globalpublichealth/s/data-availability) requires authors to make all data underlying the findings described in their manuscript fully available without restriction, with rare exception. The data should be provided as part of the manuscript or its supporting information, or deposited to a public repository. For example, in addition to summary statistics, the data points behind means, medians and variance measures should be available. If there are restrictions on publicly sharing data—e.g. participant privacy or use of data from a third party—those must be specified.

Reviewer #2: Yes

5. Is the manuscript presented in an intelligible fashion and written in standard English?

PLOS Global Public Health does not copyedit accepted manuscripts, so the language in submitted articles must be clear, correct, and unambiguous. Any typographical or grammatical errors should be corrected at revision, so please note any specific errors here.

Reviewer #2: Yes

**6. Review Comments to the Author**

Please use the space provided to explain your answers to the questions above. You may also include additional comments for the author, including concerns about dual publication, research ethics, or publication ethics. (Please upload your review as an attachment if it exceeds 20,000 characters)

1. Reviewer #2: The authors have done well in addressing my previous comments, although I have a few minor remaining comments.

   Introduction
   - This could use some more work before it is suitable for publication.
   - For example, the first paragraph is too long, suggest dividing in two and the combining the very small third paragraph in the introduction (perhaps after is too detailed in terms of the

**Response:** Modification has been made as suggested

2. Some of the information in paragraph 4 (lines 89-96) could be included in a context section in the methods (first section/paragraph in the methods) where the context of the study is described, including the currently surveillance situation for malaria

**Response:** Modification has been made as suggested.

3. combine paragraph 3 (too short and not connected to another paragraph) to paragraph 6 (lines 101-106)

**Response:** Modification has been made as suggested.

1. paragraph from lines 117-127 - also containing too much detail for an introductory paragraph, some of this information could be put into the context paragraph in the methods

**Response:** Some of the points raised in the paragraph have important context for the introduction as it highlights the problems with using only API as an indicator of malaria endemicity and the roles of other indicators in describing the true malaria burden.

2. I would reword the study aim instead to be (using a PICOT format) - to compare and contrast malaria endemicity indicators API, ABER, and SPR, in india.

**Response:** The required modification has been made as suggested.

***Methods***

3. API equation, please include reference

**Response:** Reference Added (Reference no. 12)

4. unclear why these thresholds were chosen for margins of error (5%, 15%)

**Response:** It is general convention in statistics, we take margin of error as 5%. Since recommended ABER is 10% if add and subtract margin of error from it we will have 5% to 15% range. The same was describe in manuscript “*we assume the actual prevalence of fever in the community to be ~10%, then ABER should be equal to 10% ± margin of error; if we again assume a margin of error as 5%, then ABER must range in between 5% to 15% to get comparable API and SPR.*”

***Results***

5. reasonably good (line 211) - what does this mean? moderate?

**Response:** As discussed in methods, a district where ABER values lies in between 10%±margin of error is considered in reasonable good surveillance category. If we assume ABER as 10% and margin of error as 5% then the districts where ABER is between 5% to 15% will belong to the category of good reasonable surveillance.

6. and the conjunction of SPR (line 241) - reword to be more clear

**Response:** By conjunction of SPR, we mean that we should include SPR along with API while categorisation of districts. The same was corrected in manuscript.

7. In a nutshell (line 242) - not appropriate scientific language In summary? Overall?

**Response:** Correction has been made as suggested

***Discussion***

8. Facet (line 313) - Not clear what you mean here, would suggest instead remove

**Response:** The word facet has been replaced with limitation

7. PLOS authors have the option to publish the peer review history of their article ([what does this mean?](https://journals.plos.org/globalpublichealth/s/editorial-and-peer-review-process#loc-peer-review-history)). If published, this will include your full peer review and any attached files.


**Do you want your identity to be public for this peer review?** If you choose “no”, your identity will remain anonymous but your review may still be made public.


For information about this choice, including consent withdrawal, please see our [Privacy Policy](https://www.plos.org/privacy-policy).

Reviewer #2: No

[NOTE: If reviewer comments were submitted as an attachment file, they will be attached to this email and accessible via the submission site. Please log into your account, locate the manuscript record, and check for the action link "View Attachments". If this link does not appear, there are no attachment files.]

While revising your submission, please upload your figure files to the Preflight Analysis and Conversion Engine (PACE) digital diagnostic tool, https://pacev2.apexcovantage.com/. PACE helps ensure that figures meet PLOS requirements. To use PACE, you must first register as a user. Registration is free. Then, login and navigate to the UPLOAD tab, where you will find detailed instructions on how to use the tool. If you encounter any issues or have any questions when using PACE, please email PLOS at figures@plos.org. Please note that Supporting Information files do not need this step.
